# PCLDA: An interpretable cell annotation tool for single-cell RNA-sequencing data based on simple statistical methods

**DOI:** 10.1016/j.csbj.2025.07.019

**Published:** 2025-07-23

**Authors:** Kailun Bai, Belaid Moa, Xiaojian Shao, Xuekui Zhang

**Affiliations:** a Department of Mathematics and Statistics, University of Victoria, Victoria BC, Canada; b Digital Research Alliance of Canada, Victoria BC, Canada; c Digital Technologies Research Centre, National Research Council Canada, Ottawa ON, Canada

**Keywords:** Single-cell genomics, Cell type annotation, Simple statistics, Linear discriminant analysis, Interpretable machine learning

## Abstract

Single-cell RNA sequencing (scRNA-seq) enables high-resolution analysis of cellular heterogeneity, yet accurate and consistent cell-type annotation remains a crucial challenge. Numerous automated tools exist, but their complex modeling assumptions can hinder reliability across varied datasets and protocols. We propose PCLDA, a pipeline composed of three modules: t-test-based gene screening, principal component analysis (PCA) and linear discriminant analysis (LDA), all built on simple statistical methods.

An ablation study shows that each module in PCLDA contributes significantly to performance and robustness, with two novel enhancements in the second module yielding substantial gains. Despite these additions, the model retains its original assumptions, computational efficiency, and interpretability. Benchmarking against nine state-of-the-art methods across 22 public scRNA-seq datasets and 35 distinct evaluation scenarios, PCLDA consistently achieves top-tier accuracy under both intra-dataset (cross-validation) and inter-dataset (cross-platform) conditions. Notably, when reference and query data are generated via different protocols, PCLDA remains stable and often outperforms more complex machine-learning approaches. Furthermore, PCLDA offers strong interpretability, attributed to the linear nature of its PCA and LDA modules. The final decision boundaries are linear combinations of the original gene expression values, directly reflecting the contribution of each gene to the classification. Top-weighted genes identified by PCLDA better capture biologically meaningful signals in enrichment analyses than those selected via marginal screening alone, offering deeper functional insights into cell-type specificity.

In conclusion, our work underscores the utility of carefully enhanced simple statistics methods for single-cell annotation. PCLDA's simplicity, interpretability, and consistently high performance make it a practical, reliable alternative to more complex annotation pipelines.

Code is available on GitHub:https://github.com/kellen8hao/PCLDA

## Graphical abstract

**Figure:**
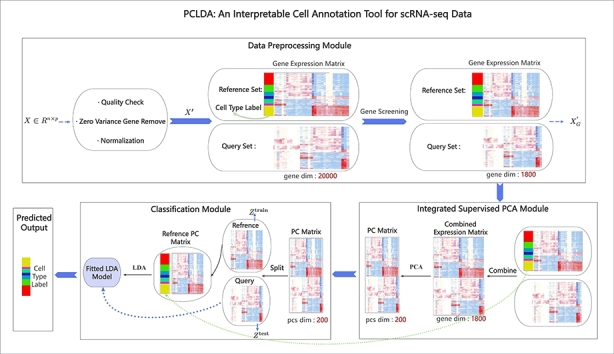

## 1 Introduction

Single-cell RNA sequencing (scRNA-seq) is a powerful high-throughput technology that enables gene expression analysis at the resolution of individual cells [1]. Unlike traditional bulk RNA sequencing, which captures the average gene expression of a mixed cell population, scRNA-seq reveals the distinct transcriptional profiles of individual cells [2]. This level of resolution is essential for identifying cell types, uncovering cellular heterogeneity, and understanding their functions, states, and lineages [3]. Therefore, scRNA-seq has been widely applied to identify the transcriptomic characteristics of various cell types within highly organized tissues and to reveal the heterogeneity and dynamics of tissues, organisms, and complex diseases [4].

Cell type annotation is the process of labeling individual cells based on their gene expression profiles and is the first and most critical step in analyzing scRNA-seq data. Accurate annotation provides a comprehensive catalog of cell types within a tissue or organism, enabling the study of cellular diversity, function, and disease mechanisms. It plays a crucial role in downstream analyses, such as reconstructing cellular pseudotimetrajectories [5], identifying cell type-specific differentially expressed genes [6], and revealing novel cell states to further understand heterogeneity [7].

Many general-purpose classification algorithms in machine learning were revised to develop cell annotation methods, including k-Nearest Neighbors [8] (e.g., scANVI [9], scClassify [10]), random forests [11] (e.g., SingleCellNet [12]), and elastic net-based models [13] (e.g., Garnett [14]), Linear Discriminant Analysis (LDA) [15] (e.g., scID [16]). More details about these automated annotation methods could be found in a recent review paper [17]. Benchmark studies were conducted to compare the performance of these annotation methods [18]. These annotation tools modified the general-purpose classification algorithms to address particular challenges in annotating cells from single-cell genomic data, making the models more complex, which requires additional model assumptions that may not hold in real-world data. So, vanilla classifiers might outperform these modified classifiers in practice. This motivates us to consider using the simplest statistical models to develop a cell annotation tool, which may not outperform complex methods in all data, but is expected to be more reliable due to its simplicity and interoperability.

We propose PCLDA, a supervised pipeline for cell type classification based on gene expression data, integrating gene screening, Principal Component Analysis (PCA), and LDA to enhance robustness and interpretability. Initially, t-statistics were used for marginal gene screening to retain the top gene sets (e.g., 400). PCA was then applied to further reduce data dimensionality, focusing on principal components that maximize class separability, rather than those that explain the highest proportion of variance. The resulting PCA scores were subsequently used as input to an LDA classifier. We compared PCLDA with various competitors in 22 real scRNA-seq data with 35 distinct evaluation scenarios and observed robust, top-tier performance of PCLDA. These results suggested thatsimple statistics often suffice and can outperform or match more complex models while mitigating the risks associated with model over-complexity.

Our contributions to the field are twofold. First, we propose a new analysis pipeline, PCLDA, which introduces two ‘novel’ modifications to the traditional PCA module. Second, through this work, we advocate for the use of simple statistics—the philosophy that classical, straightforward statistical methods can effectively solve real-world problems without unnecessary complications. Compared to complex methods, simpler methods typically involve fewer model assumptions, have fewer parameters to tune, are easier to interpret, and are often more computationally efficient and robust. Thus, when performance is comparable, the simpler approach is generally preferable. This principle is well accepted in statistical community. For example, several world-leading biostatisticians created a blog called “Simply Statistics” (https://simplystatistics.org/). They use this website to post ideas, discuss science/popular writing, highlight inspiring articles, and share advice with up-and-coming statisticians. Occam's razor [19], also known as the principle of parsimony or the law of parsimony (Latin: lex parsimoniae), is the problem-solving principle that “entities should not be multiplied beyond necessity”. Particularly, for cell annotation methods, we found that scID (modified LDA for single-cell data) was consistently outperformed by its vanilla version (LDA) in our experiments.

## 2 Method - PCLDA pipeline

**Fig. 1:**
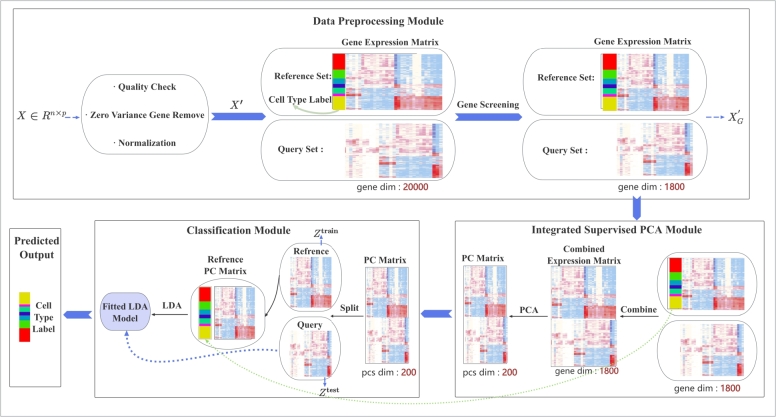
**Flowchart of the PCLDA pipeline.** The pipeline consists of three modules: (1) the *Data Preprocessing Module*, where raw gene expression data undergo normalization, log-transformation, and gene screening based on t-statistics; (2) the *Integrated Supervised PCA Module*, where principal components (PCs) are computed from concatenated reference (training) and query (test) datasets using the genes selected in the preprocessing step, and top PCs are chosen based on their supervised discriminatory ability among cell types rather than solely on explained variance; and (3) the *Classification Module (LDA Module)*, where a Linear Discriminant Analysis (LDA) model is trained using the selected PCs from the training set and then applied to classify cells in the test set. Each cell is ultimately assigned to the cell type with the highest probability according to the LDA decision function.

In this section, we describe PCLDA, a three-step pipeline for annotating single-cell data. The pipeline begins with a data preprocessing step that includes normalization, log-transformation, and gene filtering based on t-statistics. This is followed by a tailored PCA step, where supervised principal component selection is applied to the filtered data (both training and test) for dimensionality reduction. Finally, a Linear Discriminant Analysis (LDA) model is trained on the training data and used to classify cells in the test set. Fig. 1 illustrates the overall workflow, and the details of each step are described below.

**Step 1: Data Preprocessing**

Let $X\in R^{n\times p}$ be the raw gene expression matrix, where *n* is the number of cells and *p* is the number of genes. Each row corresponds to a cell, and each column corresponds to a gene. Denote by $x_{ij}$ the raw expression of gene *j* in cell *i*.

Single-cell RNA-seq data often vary widely in sequencing depth across cells. We apply log-transformed library-size normalization:

(1) $$x_{ij}^{\prime }\;=\;\log_{2}(\;1\;+\;10^{4}\;\frac{x_{ij}}{\sum_{k=1}^{p}x_{ik}}),$$

where $x_{ij}^{\prime }$ is the normalized expression for cell *i*, gene *j*. This transformation reduces technical variability and brings the data closer to a symmetric scale.

To reduce the number of genes and remove non-discriminative ones, we perform a simple t-test screening on transformed expression $x_{ij}^{\prime }$. We define a t-score to compare type *c* versus the rest:

(2) $$T_{j,c}\;=\;\frac{{\overset{¯}{x}}_{j,c}-{\overset{¯}{x}}_{j,\mathrm{rest}}}{\sqrt{\frac{s_{j,c}^{2}}{n_{c}}+\frac{s_{j,\mathrm{rest}}^{2}}{n_{\mathrm{rest}}}}},$$

where ${\overset{¯}{x}}_{j,c}$ and $s_{j,c}^{2}$ are the average expression and empirical variance of gene *j* in all cells of type *c*; ${\overset{¯}{x}}_{j,\mathrm{rest}}$ and $s_{j,\mathrm{rest}}^{2}$ are the average expression and empirical variance of gene *j* in all cells not belong to type *c*. $n_{c}$ and $n_{\mathrm{rest}}$ are the number of cells in and out of type *c*. We select, for each cell type *c*, the top *k* (usually k=300∼500) genes with highest $\left|T_{j,c}\right|$. We then pool (take the union of) these sets across all *c*.

Denote the resulting filtered gene set by *G*, with |G|≪p (often |G| is a few thousands). Let $X_{G}^{\prime }\in R^{n\times |G|}$ be the matrix of normalized log-expression restricted to these genes.

**Step 2: Dimensionality Reduction via PCA and Supervised PC Selection**

This step aims to reduce data dimensionality to minimize the risk of overfitting in LDA classifier, as well as mitigate multicollinearity by extracting orthogonal principal components (PCs). We tailored PCA in two places: PC computing and PC selection as describe below.

*PC computation:*  PCA is applied on $X_{G}^{\prime }\in R^{n\times |G|}$, the pooled data (training + testing). Unlike conventional models that apply PCA solely to the training dataset, this strategy helps capture the shared structure between the two sets, enhancing the model's ability to generalize and improve annotation performance when classifying cells not well presented in training data. Let $Z^{\text{train}},Z^{\text{test}}\in R^{n\times |G|}$ denote the resulting PC score matrices for training and test sets, respectively.

*PC selection*  The selected PC scores are used as predictors in LDA, a classifier aims to maximize ratio of the between-class variance and within-class variance. Hence, instead of selecting PCs based on the proportion of variance explained, we choose those that best separate cell types—aligning with LDA's objective. This ensures that LDA uses much fewer predictors to achieve a similarly high discriminative ratio. Specifically, PC selection is based on a score defined below.

Let $z_{i,k}$ be the *k*-th PC coordinate of cell *i* in training data. Let $\mu_{c,k}$ be the mean of $z_{i,k}$ over cells in class *c* and $\mu_{.,k}$ the overall mean of $z_{i,k}$ for all cells. Then for the *k*-th PC, the *between-class variance* and the *within-class variance* are

$${\mathrm{Var}}_{B}(z_{:,k})\;=\;\sum_{c=1}^{C}n_{c}\;{\left(\mu_{c,k}-\mu_{.,k}\right)}^{2},\;{\mathrm{Var}}_{W}(z_{:,k})\;=\;\sum_{c=1}^{C}\sum_{i\in c}{\left(z_{i,k}-\mu_{c,k}\right)}^{2}.$$

We define the ratio

(3) $$R_{k}\;=\;\frac{{\mathrm{Var}}_{B}(z_{:,k})}{{\mathrm{Var}}_{W}(z_{:,k})}.$$

A larger $R_{k}$ indicates that the *k*-th PC better separates the classes. In practice, we rank all PCs by $R_{k}$ and retain *d* top PCs with the largest values (e.g., picking the d=200 top PCs). We denote the selected *d* PCs as $Z^{\mathrm{train}}\in R^{n_{\mathrm{train}}\times d}$, which will be used as input of the next step.

**Step 3: Training Linear Discriminant Analysis (LDA) Classifier and Annotating Test Set Cells**

*Training LDA*  This step applies LDA to the selected PCs on training data $Z^{\mathrm{train}}\in R^{n_{\mathrm{train}}\times d}$, projecting the data from *d* dimension into a (C−1)-dimensional space to maximize class separability. Note C−1<<d, since the number of cell types is muchsmaller than the number of PCs we retain.

Let *W*, a *d* × (C−1) matrix, represents the projection matrix that transforms the data into a new space where the separation between different classes is maximized. LDA identifies an optimal linear combination of features that maximizes the ratio of between-class scatter to within-class scatter, thereby facilitating bothdimensionality reduction and effective classification. By enhancing inter-class separation while reducing intra-class variation, LDA serves as a robust classifier for automatic cell annotation. Specifically, LDA aims to find a transformation from data space to LDA space or a project matrix *W* by solving:

(4) $$W=\arg\max_{W}\frac{\text{tr}(W^{⊤}S_{B}W)}{\text{tr}(W^{⊤}S_{W}W)},$$

where the between-class scatter matrix $S_{B}$ and The within-class scatter matrix $S_{W}$ are defined as

$$S_{B}=\sum_{k=1}^{d}\;\sum_{c=1}^{C}n_{c}(\mu_{c,k}-\mu_{.,k}){\left(\mu_{c,k}-\mu_{.,k}\right)}^{T}e_{k}e_{k}^{T},S_{W}=\sum_{k=1}^{d}\;\sum_{c=1}^{C}\sum_{i\in c}(z_{i,k}-\mu_{c,k}){\left(z_{i,k}-\mu_{c,k}\right)}^{T}e_{k}e_{k}^{T},$$

where $e_{k}={\left[0,\ldots 0,1,0,\ldots ,0\right]}^{T}\in R^{d\times 1}$ is a unit basis vector, with all zero elements except for 1 appears in the *k*-th position. This ensures that only the *k*-th PC contributes to the scatter matrices.

*Classification of test data:*  Let $Z^{\text{test}}\in R^{n_{\text{test}}\times d}$ represent the selected PCs of test data. The prediction procedure is as follows. First, the test data is projected into the LDA space using

$$Z_{\text{LDA}}^{\text{test}}=Z^{\text{test}}W,$$

where $W\in R^{d^{\prime }\times (C-1)}$ is the LDA projection matrix.

For each class *c*, we compute the linear discriminative function

$$\delta_{c}(Z_{\text{LDA},i}^{\text{test}})=w_{c}^{T}Z_{\text{LDA},i}^{\text{test}}+b_{c},$$

where

$$w_{c}=\Sigma_{W}^{-1}\mu_{c}^{\text{LDA}},\;b_{c}=-\frac{1}{2}\mu_{c}^{{\text{LDA}}^{T}}\Sigma_{W}^{-1}\mu_{c}^{\text{LDA}}+\log P(y=c).$$

Here, the class mean $\mu_{c}^{\text{LDA}}$ and the within-class covariance matrix $\Sigma_{W}$ are computed from the training data:

$$\mu_{c}^{\text{LDA}}=\frac{1}{n_{c}}\sum_{i\in c}Z_{\text{LDA},i}^{\text{train}},$$

$$\Sigma_{W}=\frac{1}{n-C}\sum_{c=1}^{C}\sum_{i\in c}(Z_{\text{LDA},i}^{\text{train}}-\mu_{c}^{\text{LDA}}){\left(Z_{\text{LDA},i}^{\text{train}}-\mu_{c}^{\text{LDA}}\right)}^{T},$$

where $n_{c}$ is the number of training samples in cell type *c*, and *n* is the total number of training samples.

Finally, each test sample is assigned to the class that maximizes the linear discriminative function,

$${\overset{ˆ}{y}}_{i}=\arg\max_{c}\delta_{c}(Z_{\text{LDA},i}^{\text{test}}).$$

This method ensures efficient classification using a linear decision boundary in the LDA-projected space.

In summary, this three-step pipeline forms a simple yet powerful approach for single-cell annotation. Because both PCA and LDA are linear, the final decision rule for each class is essentially a linear combination of original genes. Inspecting the absolute coefficients can reveal which genes have the strongest impact on classification. In the next sections, we demonstrate its performance and compare it with state-of-the-art methods.

## 3 Results - performance evaluation using real data

### 3.1 Datasets

We compiled 22 real scRNA-seq datasets from previously published studies. These datasets differ by species (e.g. Human and Mouse), tissue contents (i.e. PBMC, pancreas, brain, etc.), and library preparation protocols (i.e. plate-based and droplet-based single-cell platforms). We used their annotated cell types as the ground truth for downstream analysis. The details of each dataset used in this study were listed as follows:

**Mouse Pancreas:** The processed mouse pancreas dataset was generated by Baron et al. [20] and downloaded from the GEO database with the accession number GSE84133. It consisted of 1886 cells and 14878 genes, and included 13 annotated cell types.

**Human Pancreas:** The human pancreas dataset was collected from five studies [20], [21], [22], [23], [24]. The processed scRNA-seq gene expression matrices can be found with their accession numbers of GSE84133, GSE85241, GSE81608, GSE86469, and E-MTAB-5061 in the GEO and EBI ArrayExpress databases, respectively. These datasets have 8562, 2285, 1492, 638 and 2394 cells, 17500, 19054, 33585, 21563, and 22939 genes as well as 13, 13, 4, 13, and 13 cell types, respectively.

**Mus musculus:** The Tabula Muris project generated two datasets of single-cell transcriptome from 20 organs and tissues of the model organism Mus musculus by using two different platforms (i.e. microfluidic droplet-based 10X Genomics and fluorescence-activated cell sorting-based SMART-Seq2) [25]. These two mouse datasets contain 24622 and 20000 cells, 22253 and 17866 genes, and 37 and 32 cell types, respectively. The datasets were downloaded through the Chan Zuckerberg Biohub https://tabula-muris.ds.czbiohub.org/.

**PBMC:** The human PBMC datasets were obtained mainly from Ding et al. [26] and Zheng et al. [27]. These datasets include four datasets generated using different versions or generations of 10X Genomics library protocols. These datasets consisted of 3362, 3222, 91649, and 2467 cells, respectively. Two of these datasets can be downloaded from the 10X website directly: https://support.10xgenomics.com/single-cell-gene-expression/datasets.

Other two are from Ding et al. [26] and can be downloaded from the GEO with the accession number of GSE132044.

Furthermore, four datasets generated using the Drop-seq, CEL-Seq2, Smart-seq2, and Seq-Well protocols in Ding et al. [26] are available with the same GEO accession number (GSE132044). These datasets consisted of 6584, 526, 526, and 3727 cells, respectively.

**Mouse Brain:** The mouse brain datasets include the primary visual cortex (PVC) and Neocortex (VISp and ALM) datasets by Tasic et al. [28], [29], and the hypothalamic arcuate-median eminence complex (HArc-ME) dataset by Campbell et al. [30], which can be downloaded from the GEO with the accession number GSE71585, GSE115746 and GSE93374. The two datasets consist of 1727, 3500 and 20921 cells, with 6, 9 and 11 cell types, respectively.

**Cell line:** The CellBench 10X and CellBench CEL-Seq2 datasets originate from the study by Tian et al. [31], are based on a mixture of five human lung cancer cell lines. We refer to them collectively as the CellBench datasets.

Using these datasets, we evaluated the performance of the methods under two scenarios: intra-dataset annotation and inter-dataset annotation. For intra-dataset annotation, five-fold cross-validation was performed on 14 datasets (listed in Table 1). This setup mimics real-world applications where reference data with consistent species, tissue composition, and library preparation protocols are available for annotating the query data. The inter-dataset annotation scenario reflects a more challenging situation, where fully consistent reference data are not available. Specifically, we examined cross-platform settings in which the reference and query datasets share the same species and tissue types but differ in their scRNA-seq library preparation protocols. To comprehensively assess this scenario, we constructed 21 reference–query dataset pairs (listed in Table 2).

**Table 1:** Datasets used for cross-validation experiments.

Datset No.	Study	Organism and Tissue	Library Platform	No. of cells
1	Baron et al [20]	Mouse pancreas	inDrop	1886
2	Baron et al [20]	Human pancreas	inDrop	8562
3	Muraro et al [21]	Human pancreas	CEL-seq2	2285
4	Segerstolpe et al [24]	Human pancreas	SMART-Seq2	2394
5	Xin et al [22]	Human pancreas	SMARTer	1492
6	Tasic et al [28]	Mouse primary visual cortex (PVC)	SMARTer	1727
7	Campbell et al [30]	Mouse HArc-ME	Drop-seq	20921
8	Ding et al [26]	Human PBMC	10x (v2)	3362
9	Schaum et al [25]	Whole Mus musculus	SMART-Seq2	24622
10	Zheng et al [27]	FACS-sorted PBMC	10X	91649
11	Zheng et al [27]	Human PBMC	10X	2467
12	Tasic et al [29]	Mouse neocortex	SMART-Seq	3500
13	Tian et al [31]	Mixture of five human cancer cell lines	CEL-seq2	909
14	Tian et al [31]	Mixture of five human cancer cell lines	10X	3918

**Table 2:** Dataset pairs for cross-platform annotation experiments.

Reference Data				Query Data			
Study	Organism and Tissue	Library Platform	No. of cells	Study	Organism and Tissue	Library Platform	No. of cells

Baron et al [20]	Human pancreas	inDrop	8562	Muraro et al [21]	Human pancreas	CEL-seq2	2285
Baron et al [20]	Human pancreas	inDrop	8562	Xin et al [22]	Human pancreas	SMARTer	1492
Muraro et al [21]	Human pancreas	CEL-seq2	2285	Xin et al [22]	Human pancreas	SMARTer	1492
Segerstolpe et al [24]	Human pancreas	SMART-Seq2	2394	Muraro et al [21]	Human pancreas	CEL-seq2	2285
Campbell et al [30]	Mouse HArc-ME	Drop-seq	20921	Tasic et al [28]	Mouse primary visual cortex	SMARTer	1727

Ding et al [26]	Human PBMC	10x(v2)	3362	Ding et al [26]	Human PBMC	10x(v3)	3222
						Drop-seq	6584
						CEL-Seq2	526
						Smart-seq2	526

Ding et al [26]	Human PBMC	10x(v3)	3222	Ding et al [26]	Human PBMC	CEL-Seq2	526
						Smart-seq2	526

Ding et al [26]	Human PBMC	Drop-seq	6584	Ding et al [26]	Human PBMC	CEL-Seq2	526
						Smart-seq2	526

Ding et al [26]	Human PBMC	inDrops	6584	Ding et al [26]	Human PBMC	10x (v2)	3362
						10x (v3)	3222
						Drop-seq	6584
						Seq-Well	3727
						CEL-Seq2	526
						Smart-seq2	526

Schaum et al [25]	Whole Mus musculus	SMART-Seq2	24622	Schaum et al [25]	Whole Mus musculus	10x	20000
Muraro et al [21]	Human pancreas	CEL-seq2	2285	Lawlor et al [23]	Human pancreas	Fluidigm C1	638

### 3.2 Experiment design

We conducted four experiments to comprehensively evaluate the performance of the PCLDA pipeline. We reported accuracy as the primary evaluation metric in the first three experiments. Accuracy was defined as the proportion of cells correctly predicted—that is, the predicted label matched the ground-truth label—out of the total number of cells.

*Experiment 1:*  To assess the sensitivity of PCLDA's parameters, we evaluated its performance under different settings by varying (i) the number of top genes retained during the gene screening step, and (ii) the number of principal components (PCs) used as input to the LDA module. Results show that PCLDA's performance remains stable as long as these parameters fall within a reasonable range.

*Experiment 2:*  To examine the contribution of each component in the PCLDA pipeline, as well as the impact of our novel modification in the PCA module, we constructed five ablation models. Three of these models were created by systematically omitting individual modules in the pipeline, while the other two replaced our PCA modification with conventional alternatives. These variants were then compared against the full PCLDA pipeline using an A/B testing framework.

*Experiment 3:*  To benchmark PCLDA's performance in both intra-dataset and inter-dataset annotation settings, we compared it with nine existing methods (listed in Table 3). These include two similarity-based methods (SingleR [32], Scmap [33]), two tree-based methods (CHETAH [34], scClassify [10]), three machine learning-based methods (SingleCellNet [12], scID [16], CaSTLe [35]), and two semi-supervised methods (SCINA [36], Seurat [37]). All competing methods were run with their default parameter settings.

**Table 3:** Overview of methods compared with the proposed PCLDA pipeline.

	Method name	Language	Computational approach
Marker gene database-based	SCINA	R	Bimodal distribution fitting to marker genes

	scmap-cell	R,web,app	Cosine distance based kNN
Correlation-based	Seurat(V5)	R	Weighted nearest neighbor
	SingleR	R	Spearman

	CHETAH	R	Classification tree
	CaSTLe	R	XGBoost classifier
Supervised classification-based	SingleCellNet	R	Random Forest
	scClassify	R, Shiny app	Weighted kNN classifier
	scID	R	Linear discriminant analysis

*Experiment 4:*  To demonstrate the interpretability of PCLDA, we performed enrichment analysis on the genes selected by the final linear model of the PCLDA pipeline, focusing on those with the largest absolute coefficients. The resulting enriched terms were compared to known biological characteristics of the annotated cell types. In addition, we compared these results to those obtained using genes selected solely by marginal screening.

### 3.3 Results

**Fig. 2:**
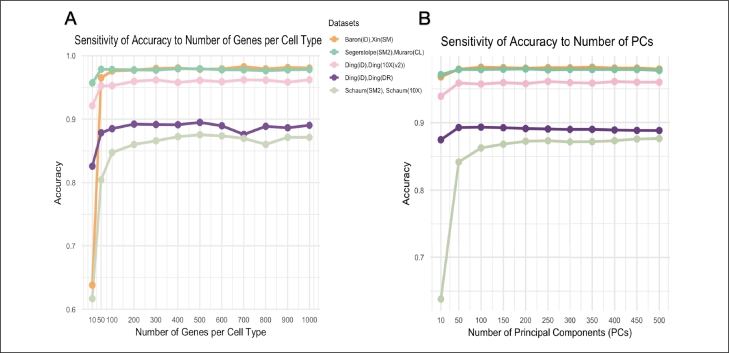
**Sensitivity analysis for PCLDA in cross-platform experiments.** (A) Effect of varying the number of genes per cell type during the gene screening step on cell-type classification accuracy across multiple datasets. Tested gene counts range from 10 to 1000. (B) Effect of varying the number of principal components (PCs) on cell-type classification accuracy across multiple datasets. Tested PC numbers range from 10 to 500. In both panels, the x-axis represents the number of genes or PCs, and the y-axis shows classification accuracy.

*Experiment 1 (i) - gene screening*  We first investigated the impact of varying the number of top genes per cell type in the gene screening step. Experiments were conducted using top 10, 50, 100, and up to 1000 genes per cell type, increasing in increments of 100. Our findings indicated that model performance remained relatively stable when using 100 or more genes (Fig. 2A). Specifically, the accuracy for the PBMC dataset (inDrop,10X(v2)) [26] was 0.952 with 50 genes, and it slightly increased to 0.96 with 200 and more genes. Similar observations were made for the Mus musculus dataset (SM2,10X) [25] and Pancreas dataset [20], [22]. For instance, the accuracy increased from 0.80 with 50 genes to 0.86 with 200 genes, and then stay with a range of 0.86 to 0.88 with more than 200 genes for the Mus musculus (SM2,10X) dataset [25]. In the Pancreas dataset, accuracy improved from 0.965 to 0.976 when comparing models with 50 genes versus 100 genes, and remained relatively stable at around 0.98 when using 100 or more genes. While accuracy gains plateaued beyond 100 genes, we selected 400 genes as the default parameter to provide additional robustness by capturing more biological variability, ensuring better generalization across different datasets. This choice balances computational efficiency with model reliability, preventing potential overfitting to a limited feature set while maintaining a manageable computational cost.

*Experiment 1 (ii) - number of PCs*  We then investigated the importance of the number of principal components (PCs) involved in the PCA Module. Experiments were performed using 10, 50, and up to 500 PCs, increasing in increments of 50, and this approach was applied across all datasets. The results show that the models achieve relatively stable performance when 50 PCs or more were used Fig. 2. Particularly, for the PBMC dataset [26] (inDrop, 10X(v2)), the accuracy was 0.959 with 50 PCs, and remained relatively stable around 0.96 as the number of PCs increased. A similar trend was observed in the Mus musculus dataset [25] (SM2,10X) and the Pancres dataset [20], [22]. For instance, in the PBMC dataset [26] (inDrop, Drop-seq), the accuracy increased from 0.875 with 10 PCs to 0.892 with 50 PCs, further improving to 0.893 with 100 PCs and stabilizing within the range of 0.890 to 0.892 when more than 100 PCs were used. For the Pancreas dataset [21], [24], accuracy improved from 0.971 with 10 PCs to 0.979 with 50 PCs and stabilized around 0.98 when using 100 or more PCs. Similarly, although accuracy gains plateaued beyond 100 PCs, we set 200 PCs as the default to ensure sufficient variance retention, capturing relevant biological signals while preventing loss of important information. This selection provides a good trade-off between computational efficiency and model robustness, optimizing performance without introducing unnecessary complexity.

*Experiment 2*  To evaluate the contribution and importance of different components in the PCLDA approach, we conducted a series of ablation experiments. The goal was to identify how each component affects the overall performance and accuracy of cell type classification. To this end, we designed five partial models, each representing a variation in the PCLDA pipeline by altering or excluding specific modules. The performance of these partial models was compared with the full PCLDA model to assess the importance of each component 3. The five partial models are as follows:

**Fig. 3:**
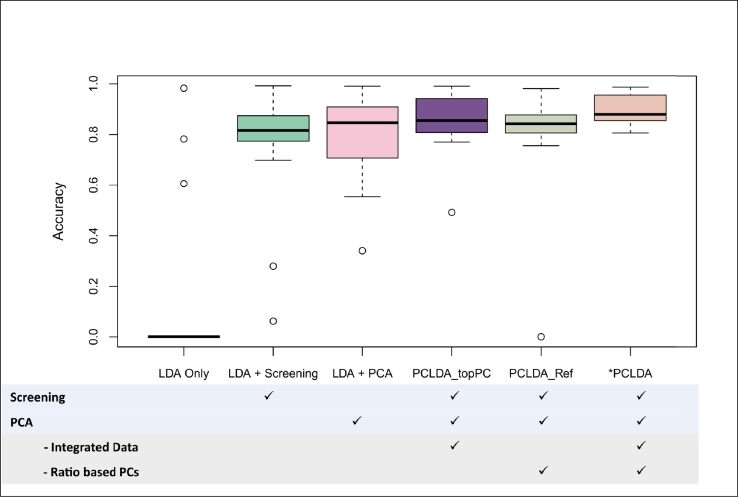
**Performance comparison across pipeline configurations.** Models were evaluated using all cross-platform datasets listed in Table 2. Six configurations were compared to assess the contribution of each component in the proposed PCLDA pipeline: (i) LDA only, (ii) LDA applied to gene-screened data, (iii) LDA applied to PCA dimension-reduced data, (iv) PCLDA_topPC (modified PCLDA using the conventional approach of selecting PCs by highest variance), (v) PCLDA_Ref (modified PCLDA using the conventional approach of applying PCA only to reference data), and (vi) PCLDA (our proposed pipeline). Paired Wilcoxon tests comparing PCLDA with each modified version confirm that the superior performance of PCLDA (as visually evident in the figure) is statistically significant (all p-values <0.002).

i) LDA Only Model: The classification was performed using the LDA model without any prior processing steps. That is, the LDA model was directly trained on the normalized scRNA-seq gene expression matrix.

ii) Gene Screening + LDA Model: A T-test-based gene screening step was applied, followed by the LDA model without dimensionality reduction. That is, no PCA based dimensionality reduction was conducted in this scenario.

iii) PCA Downscaling on Reference Data + Default PCs Selection + LDA Model: PCA was first applied to the normalized gene expression matrix of reference scRNA-seq data to reduce dimensionality. The LDA model was then trained on selected top PCs. That is, no gene screening was conducted in this scenario.

iv) Gene Screening + PCA Downscaling on Integrative Data + Default PCs Selection + LDA Model: A T-test-based gene screening step was applied on the reference data, followed by PCA for dimensionality reduction, where the default number of top PCs was selected on the integrated dataset (including both reference and query data). Finally, the LDA model was trained on the reduced PC space using the reference data.

v) Gene Screening + PCA on Reference Data Only + Variance Ratio PCs Selection + LDA Model: A T-test-based gene screening step was applied, followed by PCA downscaling applied exclusively to the reference dataset. The number of PCs was determined based on the variance ratio criterion. Finally, the LDA model was trained using the selected PCs from the reference data for classification.

We observed that the full PCLDA model consistently achieved the highest and most stable accuracy across datasets. Notably, using the LDA model alone (model i) almost always resulted in out-of-memory errors, underscoring its inability to handle the high dimensionality of scRNA-seq data. Introducing either gene screening (model ii) or PCA (model iii) resolved these memory issues by reducing dimensionality, confirming the necessity of a preprocessing step before applying LDA.

Further, models iv (PCLDA_topPC) and v (PCLDA_Ref) outperformed models ii and iii, indicating that combining gene screening and PCA is more effective than applying either method alone. This is likely because the two techniques serve different but complementary purposes: gene screening removes irrelevant predictors, while PCA transforms the data into orthogonal components. Preserving both steps helps ensure that informative structure is retained while mitigating multicollinearity and overfitting.

Finally, our full PCLDA model (model vi) significantly outperformed all alternatives (all paired Wilcoxson p-values <0.002), including models iv and v. This demonstrates the value of our two novel modifications to the PCA module: (1) performing PCA jointly on the concatenated reference and query datasets, which improves alignment and reduces batch effects; and (2) selecting principal components using a supervised criterion that prioritizes cell-type discriminative signals over variance explained. Together, these enhancements led to improved accuracy and more robust performance across all cross-platform datasets.

*Experiment 3*  We then compared the classification accuracy of PCLDA with that of other peer methods on the above-mentioned scRNA-seq datasets, covering both intra-dataset and inter-dataset scenarios.

**Fig. 4:**
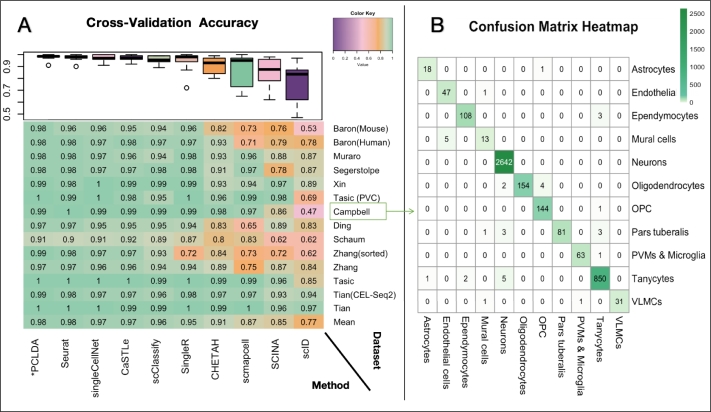
**Performance evaluation using cross-validation experiments.** (A) Heatmap comparing the accuracy of PCLDA and nine other cell-type annotation methods across 14 cross-validation experiments (in the same order as Table 1). Rows represent individual experiments, with the bottom row indicating the average accuracy of each method. Columns represent the ten methods, ordered left-to-right by their average accuracy. A boxplot above the heatmap summarizes the distribution of accuracies for each method across the 14 experiments. (B) Detailed annotation results from PCLDA on the mouse brain dataset profiled by Drop-seq, demonstrating near-perfect performance.

We first tested the classification accuracy of nine methods listed in Table 3 on fourteen publicly available scRNA-seq datasets (Table 1). These datasets include three peripheral blood mononuclear cell (PBMC) datasets, four human pancreatic islet datasets, one mouse pancreas dataset, one Mus musculus dataset, three mouse brain datasets and two cell lines datasets. The detailed comparison results were shown in Fig. 4. It demonstrated that most of the methods consistently showed strong performance across all datasets. Notably, PCLDA, Seurat, and singleCellNet, CaSTLe achieved remarkable average accuracies of 0.98, 0.98, 0.97 and 0.97, respectively. In contrast, some methods, like scID, SCINA, scmapcell exhibited poor performance (average accuracy < 0.87). Additionally, other methods including scClassify, SingleR, and CHETAH performed decently well on most datasets but struggled with the Mus musculus data and PBMC datasets. It is worth emphasizing that the Mus musculus dataset from the Smart-Seq2 platform contains 37 cell types 1, and certain cell types have a very limited number of cells (very unbalanced dataset), making the classification task more challenging. As a result, those methods struggled to achieve high performance on the Mus musculus dataset. Overall, in the intra-dataset cross-validation scenario, the PCLDA is ranked top one in most of the datasets and has high stability with no outliers. Particularly, when dealing with large PBMC dataset (Zhang (FACS-sorted)), which have 90k+ cells, the PCLDA achieved the top one performance, indicating that PCLDA is very effective in handling large data.

**Fig. 5:**
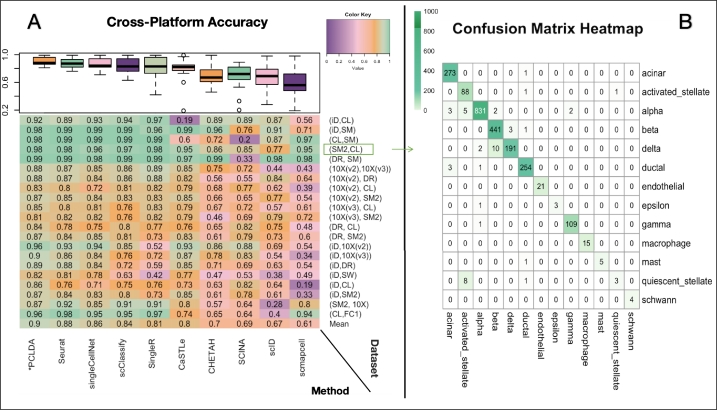
**Performance evaluation using cross-platform (external validation) annotation experiments.** (A) Heatmap comparing the annotation accuracy of PCLDA with nine competing methods under cross-platform scenarios. Methods are sorted from left to right based on their average accuracy across datasets. Rows represent matched reference-query dataset pairs that differ only by sequencing protocols (listed in the same order as Table 2). The heatmap follows the format of Fig. 4, but row labels here indicate the protocol pairs (reference–query), abbreviated as follows: iD = inDrops; CL = CEL-Seq2; SM = SMARTer; SM2 = Smart-seq2; DR = Drop-seq; 10X(v2) = 10x Chromium (v2); 10X(v3) = 10x Chromium (v3); SW = Seq-Well; FC1 = Fluidigm C1. A boxplot above the heatmap summarizes the distribution of accuracies for each method across all experiments. (B) Detailed annotation performance of PCLDA on human pancreas datasets, using SMART-seq2 as the reference and CEL-Seq2 as the query.

To assess the annotation tools in a different scenario from the previous intra-dataset cross-validation, we conducted an inter-dataset performance evaluation using 21 datasets pairs, as shown in Table 2. While not all the models perform well on all the datasets, a few methods (e.g. scID and scmapcell) even showed poor performance in most comparisons with accuracies lower than 0.7. In contrast, the PCLDA model consistently achieved the top performance and demonstrated the top one rank in all 21 comparisons, along with Seurat and singleCellNet (Fig. 5). The average accuracy for PCLDA, Seurat and singleCellNet were 0.9, 0.88 and 0.86, respectively.

To further evaluate performance under cross-platform scenarios, we computed a confidence score for each cell type by averaging the predicted probabilities of all cells assigned to that type. This score reflects the model's certainty in its predictions. Results are summarized in Table S5, where NaN or NA values indicate cell types that were not present in the test set.

Altogether, the results across the 35 benchmark experiments in the two scenarios demonstrated that the performance of PCLDA is robust and stable. The model performance remains consistently high for both intra-dataset prediction (cross-validation scenario) and inter-dataset prediction (cross-protocol scenario).

To further evaluate model generalizability, we assessed the ability of cell annotation methods to reject or correctly handle previously unseen cell types. We designed an additional series of cross-platform classification tasks in which the query dataset includes cell populations absent from the reference. This setup reflects a realistic challenge in scRNA-seq annotation, where biological diversity in new samples may exceed that represented in existing reference datasets. A summary of the evaluation results is included in the Supplementary Material, with a detailed dataset summary (Table S2), accuracy metrics (Table S3), unknown prediction rates (Table S4), and confusion matrices (Table S6).

*Experiment 4 - enrichment analysis*  We observe that PCLDA not only enhances cell type classification but also provides an interpretable framework for identifying the most influential genes in the classification process. Since both the PCA step and the LDA step are linear models, PCLDA's final classification model also makes decision based on linear combination of expression level of original genes. Hence, by examining the weights assigned to each gene in the PCLDA model, we can determine which genes play a significant role in distinguishing between cell types. This allows us to pinpoint key genes that drive the final classification, offering valuable insight into their potential biological relevance and functional roles in cell-type specificity.

**Fig. 6:**
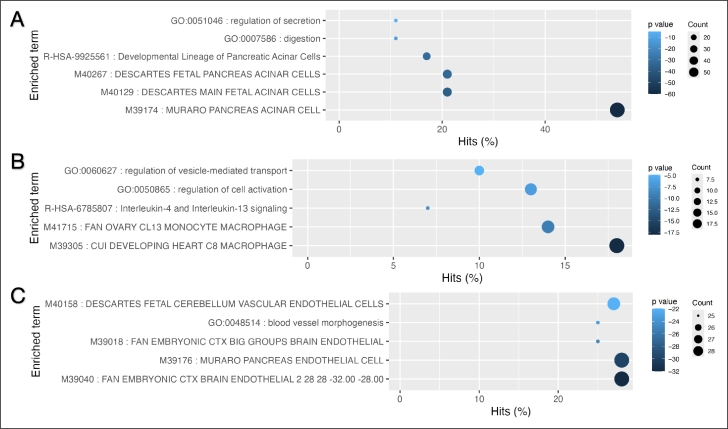
**Enriched GO terms for the different cell type related top 100 genes**: (A) Enriched GO terms for the top 100 genes associated with Acinar cells. (B) Enriched GO terms for the top 100 genes associated with Macrophages. (C) Enriched GO terms for the top 100 genes associated with Endothelial cells.

To further investigate how the genes identified by PCLDA compare to those selected through marginal screening, we performed an enrichment analysis. By comparing these gene sets, we aim to determine whether PCLDA selects distinct or overlapping genes, providing a deeper understanding of the genes that drive cell-type classification. This analysis not only helps us evaluate the effectiveness of PCLDA in capturing biologically relevant signals but also enables us to explore functional insights. Through functional enrichment, we can further investigate the biological roles of these genes and their potential contribution to defining cell-type specificity and underlying mechanisms. To achieve this, we selected the top 100 genes for each cell type from both the PCLDA-weighted gene list and the gene screening-derived gene list. We then perform the enrichment analysis using Metascape [38] on the selected top 100 genes per cell type. Our results indicate the enriched terms (e.g. functional pathways and cell type specific signatures) are highly relevant with the corresponding input cell type. For example, in the Human Pancreas dataset (Baron et al.) [20], the top genes associated with acinar cell were linked to acinar cell receptor signaling pathways. Moreover, these genes are also significantly enriched in previously published cell type signatures of acinar cell, see the Fig. 6.

Similarly, the top genes in Macrophage were observed to be enriched at the Interleukin-4 and Interleukin-13 signaling pathway and CUI DEVELOPING HEART C8 MACROPHAGE cells. The IL-4 and IL-13 signaling pathways are known to be integral to macrophage biology, influencing their activation states, functional roles, and responses to environmental stimuli. Additionally, Endothelial cell-related top genes were enriched at the pathways associated with blood vessel morphogenesis. These top genes were also significantly enriched at the endothelial cell type-specific signal genes that were reported from multiple studies. See Fig. 6 for the details.

To further evaluate the effectiveness of PCLDA in identifying cell-type-specific genes, we conducted enrichment analysis on the top 100 genes selected from the gene screening step for each corresponding cell type. While a few common or functionally similar items were observed, the genes identified by PCLDA exhibited significantly stronger enrichment for cell-type signatures, as reflected by both the number (percentage) of counts in the “Cell Type Signatures” term and their corresponding log10(q) values. For example, among the top 100 genes identified by PCLDA for the acinar cell type, 54% overlapped with the reported pancreas acinar cell signatures from Muraro et al. [21], compared to only 27% overlap for the top 100 genes selected via the gene screening step. An even stronger enrichment was observed for both endothelial and macrophage cells. In particular, 28% of the top 100 genes from PCLDA overlapped with pancreas endothelial cell type signatures from Muraro et al. [21] whereas no reported endothelial cell type signatures terms are detected for the top 100 genes from the gene screening step. Similarly, 18% of the top genes from PCLDA are reported as cell type signatures of macrophage from Cui et al. [39], while no macrophage cell type signature terms are enriched for the top genes obtained from the gene screening step. These results underscore the ability of PCLDA to capture biologically relevant cell-type-specific gene signatures more effectively compared to traditional gene screening methods.

## 4 Discussion

In this study, we proposed an analysis pipeline, PCLDA, for single cell annotation using simple statistics. Compared to other complex statistical and machine learning models, the proposed simple statistics model achieved comparable performance to the top ones for cell annotation tasks with reference and query datasets generated using the same or different protocols. In most cases, annotating cell types using reference data from the same protocol is ideal and our proposed model shows excellent performance for such cases. Even in the other cases where the same protocol reference data is not available, our model showed outstanding robustness and reliability, supporting our hypothesis that simple models are generally much more reliable.

*Novelty*  In addition to proposing a novel analysis pipeline, our primary innovations are introduced within the Integrated Supervised PCA Module. In scRNA-seq analysis, PCA is a widely used technique for dimensionality reduction. Traditionally, PCA is performed separately on individual datasets, and PCs are selected based on their explained variance. While effective in many contexts, these conventional approaches may not fully address certain challenges inherent in scRNA-seq data integration and cell-type discrimination.

First, performing PCA jointly on the concatenated reference (training) and query (test) datasets offers significant advantages in aligning feature spaces and mitigating batch effects. This approach facilitates the identification of shared structures between datasets, enhancing the integration process. By applying PCA to the combined dataset, the resulting PCs capture variations common to both datasets, leading to more accurate downstream analyses.

Second, selecting PCs based solely on explained variance may not effectively capture biologically relevant variations, especially those pertinent to distinguishing cell types. A supervised selection criterion that evaluates the ratio of variance across different cell types can prioritize PCs that are more informative for classification tasks. The criterion directly mirrors the optimization objective of LDA, allowing the LDA classifier to achieve enhanced discrimination power with fewer and more informative PCs.

*Robustness and simplicity*  A distinguishing feature of PCLDA is its use of straightforward yet powerful components including t-tests, PCA, and LDA. These methods are computationally efficient, well understood, and less prone to overfitting than more complex models. This simplicity enhances robustness, making PCLDA effective across diverse datasets and experimental conditions. Additionally, its linear structure ensures that the final results are biologically interpretable. In contrast, other LDA-based methods, such as scID, which employs a weighted LDA approach, exhibited varying levels of accuracy in our cross-protocol comparisons. As demonstrated in our computational experiments, increasing model complexity or the number of parameters does not necessarily result in improved annotation performance, especially when experimental protocols differ between the reference and query datasets. This suggests that simpler and well-structured approaches, such as PCLDA, can provide more consistent performance under these challenging scenarios.

*Interpretability and biological insights*  A key strength of PCLDA lies in its interpretability. Because both PCA and LDA are linear methods, the final decision rule in PCLDA is also a linear combination of the original genes. This means that each gene contributes directly to the final classification decision with a weight (or coefficient) that is derived from the PCA and LDA transformations.

The coefficients of genes in the final rule provide two critical insights: 1. Effect direction: The sign of a gene's coefficient in the PCLDA model reflects its role in distinguishing a specific cell type from others. A positive coefficient indicates that the gene is more highly expressed in the target cell type relative to other cell types, making it a potential marker gene for classification. Conversely, a negative coefficient suggests that the gene is expressed at a lower level in the target cell type compared to others, potentially indicating its relative depletion in that cell type. This allows researchers to infer the biological role of genes in distinguishing between cell types. 2. Effect magnitude: The magnitude of a gene's coefficient reflects its relative importance in the decision-making process. Genes with larger absolute coefficients contribute more strongly to the classification outcome, whereas those with smaller coefficients have a weaker influence on distinguishing between cell types.

By standardizing gene expression data before applying PCLDA, the coefficients become directly comparable across genes. This standardization ensures that the magnitude of the coefficients is a meaningful measure of their importance, providing quantitative insights into the contribution of each gene to the classification task. As a result, PCLDA offers not only robust predictions but also biologically interpretable models, enabling researchers to identify key regulators of cell type differences and understand their functional roles. This interpretability makes PCLDA particularly attractive for applications in biology, where understanding the mechanisms underlying classification decisions is often as important as achieving high predictive accuracy.

## 5 Conclusion

In this study, we proposed PCLDA, a simple yet effective pipeline for cell-type annotation using single-cell RNA sequencing data. PCLDA is built upon well-established statistical methods—t-tests, PCA, and LDA—augmented with two key innovations: (1) performing PCA jointly on concatenated reference and query datasets to better align data and reduce batch effects, and (2) selecting principal components using a supervised criterion that prioritizes cell-type discriminative features rather than relying solely on explained variance. These modifications significantly enhance the interpretability, robustness, and classification accuracy of the pipeline. Through extensive experiments, we demonstrated that PCLDA performs competitively with or surpasses more complex machine learning-based methods across both cross-validation and cross-protocol scenarios. Notably, it maintains stable performance even when the number of cell types increases or when reference and query datasets differ in library preparation protocols. Its linear, interpretable structure also makes it more accessible and transparent for biological researchers.

In conclusion, PCLDA provides a practical and reliable alternative for cell-type annotation, emphasizing that carefully enhanced simple statistics methods can offer performance comparable to state-of-the-art models while retaining interpretability, computational efficiency, and ease of use.

## Funding

This work is supported in part by funds from 10.13039/501100000233Genome BC Sector Innovation Program (X.Z.), 10.13039/501100000046NRC Digital Health and Geospatial Analytics Program (X.Z., X.S.), and the 10.13039/501100001804Canada Research Chairs (CRC-2021-00232 X.Z.), 10.13039/501100000245Michael Smith Foundation for Health Research (SCH-2022-2553 X.Z.).

## CRediT authorship contribution statement

**Kailun Bai:** Writing – review & editing, Writing – original draft, Visualization, Software, Project administration, Methodology, Formal analysis, Data curation. **Belaid Moa:** Writing – review & editing, Methodology, Investigation. **Xiaojian Shao:** Writing – review & editing, Supervision, Project administration, Methodology, Investigation, Funding acquisition, Formal analysis. **Xuekui Zhang:** Writing – review & editing, Supervision, Project administration, Methodology, Investigation, Funding acquisition.

## Declaration of Competing Interest

The authors declare that they have no known competing financial interests or personal relationships that could have appeared to influence the work reported in this paper.

## Appendix A Supplementary material

MMC 1 Supplementary Document S1-S4, Supplementary Table1-4, Supplementary Figure 1. MMC 1

MMC 2 Supplementary Table S5-6. MMC 2
