# Supplementary material for: PCLDA: An interpretable cell annotation tool for single-cell RNA-sequencing data based on simple statistical methods
Source: Comput Struct Biotechnol J. 2025 Jul 23;27:3264–74. doi: 10.1016/j.csbj.2025.07.019 (PMC12329077; doi:10.1016/j.csbj.2025.07.019)
Supplement: MMC 1 — Supplementary Document S1-S4, Supplementary Table1-4, Supplementary Figure 1. [file mmc1.pdf]

PCLDA: An interpretable cell annotation tool for single-cell RNA-sequencing data based on simple statistical methods

Kailun Bai[1], Belaid Moa[2], Xiaojian Shao[1,3*], and Xuekui Zhang[1**]

[1] Department of Mathematics and Statistics, University of Victoria, Victoria BC, Canada
[2] Digital Research Alliance of Canada, Victoria BC, Canada
[3] Digital Technologies Research Centre, National Research Council Canada, Ottawa ON, Canada

S1 PCLDA: Variance ratio based PC selection

Let L be an LDA model. L can be viewed as finding the vectors that maximize the between classes variance compared to within classes variance:

$$\max_{\theta} L(\theta),$$

where

$$L(\theta) = \frac{\theta^T \Sigma_B \theta}{\theta^T \Sigma_W \theta}.$$

PCA, on the other hands, finds the vectors that maximizes the variance across the samples:

$$\max_{\theta} P_T(\theta),$$

where

$$P_T(\theta) = \frac{\theta^T \Sigma_T \theta}{\theta^T \theta}.$$

Where Σ_T is the total variance between samples, Σ_B is the between classes variance, and Σ_W is within-classes variance. These are also known as scattering matrices. If we consider X to be the data matrix with N rows as observations (cells in our case) and K columns as features (pcs), then these scattering matrices are related as follows:

$$\begin{aligned} \Sigma_T &= (X - \mu)^T (X - \mu) \\ &= \left((X_i - \mu_i)^T (X_j - \mu_j)\right)_{ij}, \end{aligned}$$

where X_i and X_j are the ith and j^{th} columns of X, respectively, M^T denotes the transpose of a matrix/vector M, and $\mu = (\mu_1, \ldots, \mu_K)$ is the mean vector of X across the rows. If we assume the cells are sorted according to their cell types and express each column in terms of the blocks associated with these types, we can write

* Corresponding authors: xiaojian.shao@nrc-cnrc.gc.ca
** Corresponding authors: xuekui@uvic.ca

the expression above as:

$$\begin{aligned}
\Sigma_T &= \big((X_i-\mu_i)^T(X_j-\mu_j)\big)_{ij} \\
&= \left(\sum_{p=1}^{C}(X_i^p-\mu_i)^T(X_j^p-\mu_j)\right)_{ij} \\
&= \left(\sum_{p=1}^{C}(X_i^p-\mu_i^p+\mu_i^p-\mu_i)^T(X_j^p-\mu_j^p+\mu_j^p-\mu_j)\right)_{ij} \\
&= \left(\sum_{p=1}^{C}(X_i^p-\mu_i^p)^T(X_j^p-\mu_j^p)+\sum_{p=1}^{C}(\mu_i^p-\mu_i)^T(X_j^p-\mu_j^p)\right. \\
&\quad \left.+\sum_{p=1}^{C}(X_i^p-\mu_i^p)^T(\mu_j^p-\mu_j)+\sum_{p=1}^{C}N_p(\mu_i^p-\mu_i)(\mu_j^p-\mu_j)\right)_{ij} \\
&= \Sigma_B+\Sigma_W
\end{aligned}$$

From the expression above, we have

$$\Sigma_B = \Sigma_T - \Sigma_W.$$

If we substitute Σ_B with this expression in the LDA model, we deduce that the LDA expression is equivalent to:

$$L(\theta) = \frac{\theta^T\Sigma_T\theta - \theta^T\Sigma_W\theta}{\theta^T\Sigma_W\theta} = \frac{\theta^T\Sigma_T\theta}{\theta^T\Sigma_W\theta} - 1.$$

We therefore see that

$$L(\theta) = \frac{P_T(\theta)}{P_W(\theta)} - 1, \tag{1}$$

where

$$P_W(\theta) = \frac{\theta^T\Sigma_W\theta}{\theta^T\theta} = \sum_{p=1}^{C}\frac{\theta^T\Sigma_{W_i}\theta}{\theta^T\theta} = \sum_{p=1}^{C}P_{W_i}(\theta),$$

where Σ_{W_i} is the within class scattering matrix of cell type i.

The expression $P_W(\theta)$ expresses the PCA model for within classes that should be minimized instead of maximized.

We therefore see that PCA seeks a general linear representation of the data in a lower dimension without using any extra information, while LDA seeks the same thing but with minimizing the variances within the classes.

This give us a nice way to assess the performance of PCA in a classification algorithm, which is to check $P_{W_i}(\theta_k)$ for all eigenvectors θ_k computed from $\max_\theta P_T(\theta)$. A θ_k that drastically increases $P_{W_i}(\theta_k)$ for any class should be handled with caution. That also could be a nice criteria to choose the number of PCs to use for classification. This allows us to turn PCA into a semi-supervised PCA with takes into account the information about the classes.

Another way to make use of the above results is to compute $P_W(\theta_k)$ for eigenvectors θ_k and add the ones with min P_W to the list of PCs.

S2 F1 Score Comparison results for Cross Platform Datasets

In FigureS1. The Micro F1 Score of PCLDA and 9 competing methods in the cross-platform scenario. The methods were sorted by their average performance across the datasets, ranging from average F1 Score of 0.90 to 0.61.

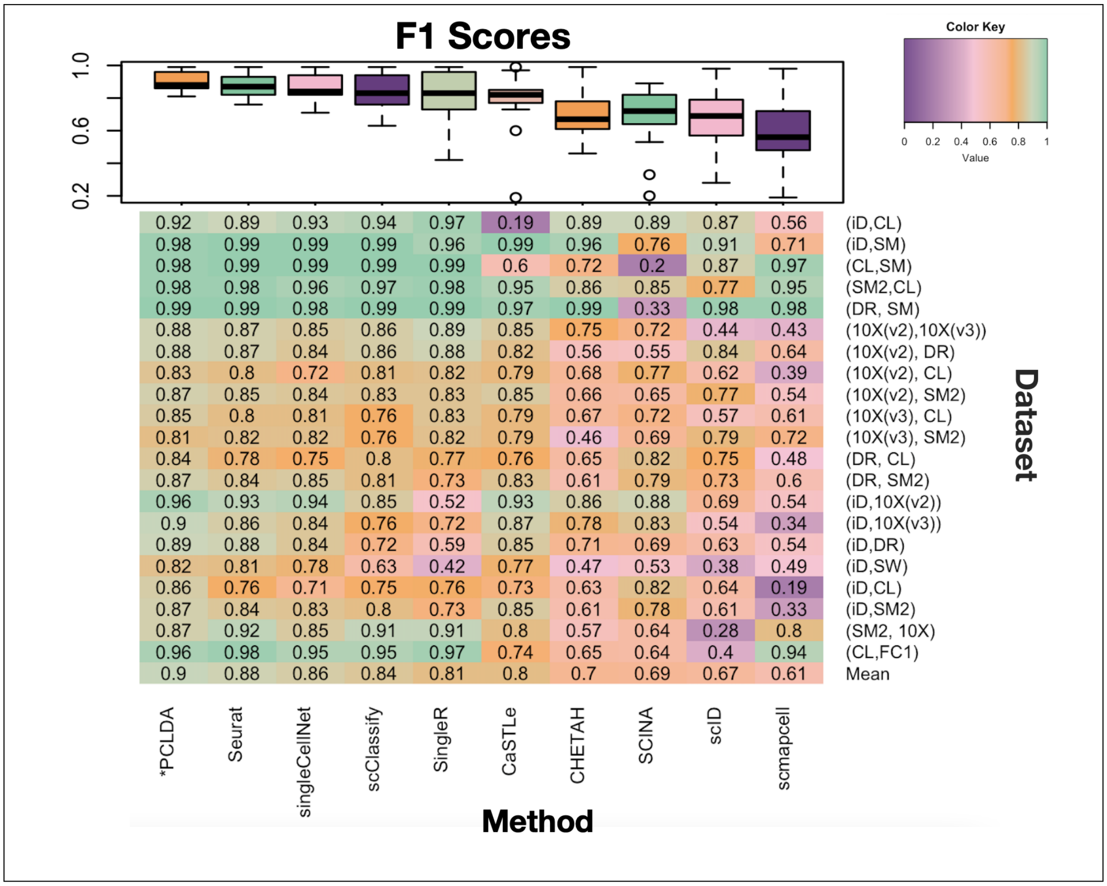

Fig. S1: Performance evaluation of cross-platform annotation using F1 Scores. The F1 score accuracy of PCLDA and 9 competing methods in the cross-platform scenario were presented. The methods were sorted by their average performance (listed as the bottom row) across the datasets. Reference and query datasets are well matched, differing only in the library protocols (rows displayed in the same order as in Table 2). Row names show the pair of two protocols in reference and query sets. The protocol names are abbreviated as iD= inDrops; CL= CEL-Seq2; SM= SMARTer; SM2= Smart-seq2; DR= Drop-seq; 10X(v2)= 10x Chromium (v2); 10X(v3)= 10x Chromium (v3); SW= Seq-Well; FC1= Fluidigm C1.

S3 Enriched GO terms for the Acinar-cell related top 100 genes from gene screening

Table S1: Enriched GO terms for the Acinar-cell related top 100 genes from gene screening

GO	Category	Description	Count	%	Log10(P)	Log10(q)
R-HSA-9925561	Reactome Gene Sets	Developmental Lineage of Pancreatic Acinar Cells	9	9.00	-13.98	-9.64
M39174	Cell Type Signatures	MURARO PANCREAS ACINAR CELL	27	27	-20	-17
M40129	Cell Type Signatures	DESCARTES MAIN FETAL ACINAR CELLS	14	14	-22	-18
M40267	Cell Type Signatures	DESCARTES FETAL PANCREAS ACINAR CELLS	13	13	-18	-14

S4 Cross-platform Evaluation with Unseen Cell Types

To evaluate the capacity of cell annotation methods to handle previously *unseen* cell types, we designed a series of cross-platform classification tasks where the query dataset contains cell types that are absent from the reference dataset. These scenarios mimic realistic challenges in single-cell integration, particularly when the biological diversity of the query exceeds that of the labeled reference.

We curated 12 dataset pairs from human PBMC and mouse brain data, spanning multiple sequencing protocols such as CEL-Seq2, Smart-seq2, SMARTer, 10x Chromium (v1/v2/v3), Drop-seq, inDrops, and Seq-Well. For each pair, the reference and query datasets were chosen to differ in both technical platforms and biological composition. Table S2 summarizes all dataset pairs used in this evaluation, including platform, number of cells, and number of cell types.

To benchmark annotation performance under these challenging conditions, we first computed the accuracy of each method across all 12 dataset pairs. As shown in Table S3, `PCLDA` consistently achieved high performance, with average accuracy exceeding 0.79. Several other methods, such as Seurat and SingleR, also performed well in select tasks, while methods like CHETAH and SCINA exhibited highly variable results.

To further interpret performance under the presence of novel cell types, we examined the proportion of cells each method labeled as "unknown," which reflects its rejection capability. As shown in Table S4, methods such as SCINA, CHETAH, and scID returned moderate to high unknown rates, particularly in dataset pairs with substantial platform or population divergence. In contrast, PCLDA, which is based on supervised discriminant learning, assigned all cells to known classes by design and consistently yielded zero unknown predictions. This outcome may be attributed to the relatively small proportion of novel cell types in the query datasets, limiting the observable benefit of unknown rejection.

To provide a more comprehensive evaluation, we also generated 2×2 confusion matrices comparing known versus unknown predictions for each method across all 12 dataset pairs. These matrices offer detailed insights into how effectively each tool distinguishes between known and novel cell populations. The results are provided in Supplementary Table S6.

Together, the accuracy scores (Table S3), unknown prediction rates (Table S4), and confusion matrix (Table S6) analysis collectively illustrate the strengths and limitations of each method in handling novel cell types during cross-platform annotation.

Table S2: Summary of dataset pairs for evaluation with unseen cell types in the query data

**Reference Data**						**Query Data (with unseen cell types)**					
Study	Organism and Tissue	Platform	No. of Cells	No. of Cell Types	GEO/EBI Accession	Study	Organism and Tissue	Platform	No. of Cells	No. of Cell Types	GEO/EBI Accession
Ding et al	Human PBMC	CEL-Seq2	526	7	GSE132044	Ding et al	Human PBMC	10x Chromium (v2)	3362	9	GSE132044
						Ding et al	Human PBMC	10x Chromium (v3)	3222	8	
						Ding et al	Human PBMC	Seq-Well	3727	7	
Ding et al	Human PBMC	Smart-seq2	526	7	GSE132044	Ding et al	Human PBMC	10x Chromium (v2)	3362	9	GSE132044
						Ding et al	Human PBMC	10x Chromium (v3)	3222	8	
						Ding et al	Human PBMC	Drop-seq	6584	9	
						Ding et al	Human PBMC	inDrops	6584	9	
						Ding et al	Human PBMC	Seq-Well	3727	7	
Tasic et al	Mouse PVC	SMARTer	1727	6	GSE71585	Tasic et al	Mouse PVC	SMART-seq v4	13825	9	GSE115746
						Tasic et al	Mouse ALM	SMART-seq v4	8288	9	GSE115746
						Campbell et al	Mouse HArc-ME	Drop-seq	20921	11	GSE93374
						Hochgerner et al	Mouse hippocampus	10x Chromium (v1)	5262	22	GSE95315

Table S3: Benchmarking results across 12 dataset pairs with unseen cell types in query data

Dataset Pair	PCLDA	Seurat	SingleR	scClassify	singleCellNet	CaSTLe	scmap-cell	scID	SCINA	CHETAH
CEL-Seq2 → 10x Chromium (v2)	0.79	0.87	0.86	0.83	0.78	0.69	0.66	0.71	0.49	0.36
CEL-Seq2 → 10x Chromium (v3)	0.77	0.67	0.67	0.71	0.40	0.55	0.48	0.49	0.28	0.23
CEL-Seq2 → Seq-Well	0.73	0.72	0.72	0.72	0.71	0.68	0.72	0.43	0.87	0.92
Smart-seq2 → 10x Chromium (v2)	0.83	0.78	0.78	0.78	0.71	0.72	0.75	0.38	0.58	0.73
Smart-seq2 → 10x Chromium (v3)	0.84	0.89	0.88	0.84	0.80	0.80	0.64	0.70	0.65	0.50
Smart-seq2 → Drop-seq	0.75	0.84	0.82	0.79	0.66	0.55	0.57	0.77	0.33	0.26
Smart-seq2 → inDrops	0.59	0.68	0.72	0.65	0.50	0.25	0.26	0.36	0.34	0.08
Smart-seq2 → Seq-Well	0.71	0.74	0.79	0.72	0.48	0.38	0.22	0.51	0.36	0.15
SMARTer → SMART-seq v4 (PVC)	0.99	0.89	0.78	0.86	0.77	0.78	0.74	0.76	0.52	0.46
SMARTer → SMART-seq v4 (ALM)	0.98	0.87	0.88	0.83	0.77	0.81	0.74	0.60	0.66	0.65
SMARTer → Drop-seq (HArc-ME)	0.72	0.83	0.75	0.82	0.65	0.68	0.67	0.77	0.38	0.50
SMARTer → 10x Chromium (v1)	0.76	0.72	0.82	0.83	0.48	0.58	0.47	0.42	0.33	0.14
Mean	0.79	0.79	0.79	0.78	0.64	0.62	0.58	0.57	0.48	0.42

Table S4: Proportion of unknown predictions in cross-platform tasks

Dataset Pair	PCLDA	CaSTLe	CHETAH	scClassify	scID	SCINA	scmap-cell	Seurat	singleCellNet	SingleR
CEL-Seq2 → 10x Chromium (v2)	0.00	0.00	0.61	0.00	0.11	0.44	0.07	0.00	0.12	0.00
CEL-Seq2 → 10x Chromium (v3)	0.00	0.00	0.45	0.00	0.21	0.28	0.08	0.00	0.05	0.00
CEL-Seq2 → Seq-Well	0.00	0.00	0.91	0.03	0.11	0.56	0.51	0.00	0.41	0.00
Smart-seq2 → 10x Chromium (v2)	0.00	0.00	0.53	0.01	0.07	0.41	0.03	0.00	0.12	0.00
Smart-seq2 → 10x Chromium (v3)	0.00	0.00	0.23	0.00	0.29	0.22	0.06	0.00	0.06	0.00
Smart-seq2 → Drop-seq	0.00	0.00	0.47	0.01	0.09	0.51	0.11	0.00	0.21	0.00
Smart-seq2 → inDrops	0.00	0.00	0.75	0.02	0.04	0.63	0.35	0.00	0.52	0.00
Smart-seq2 → Seq-Well	0.00	0.00	0.88	0.02	0.31	0.61	0.40	0.00	0.47	0.00
SMARTer → SMART-seq v4 (PVC)	0.00	0.00	0.00	0.05	0.26	0.02	0.00	0.00	0.01	0.00
SMARTer → SMART-seq v4 (ALM)	0.00	0.00	0.00	0.06	0.65	0.38	0.00	0.00	0.01	0.00
SMARTer → Drop-seq (HArc-ME)	0.00	0.00	0.28	0.00	0.58	0.35	0.05	0.00	0.56	0.00
SMARTer → 10x Chromium (v1)	0.00	0.00	0.09	0.00	0.81	0.60	0.04	0.00	0.44	0.00
